# Case report: solitary splenic metastasis occurring 19 months after primary treatment for occult breast cancer

**DOI:** 10.3389/fonc.2022.957490

**Published:** 2022-07-28

**Authors:** Yuanqi Chen, Liulu Zhang, Taotao Sun, Min-Yi Cheng, Jiachen Zou, Kun Wang

**Affiliations:** ^1^ The Second School of Clinical Medicine, Southern Medical University, Guangzhou, China; ^2^ Department of Breast Cancer, Cancer Center, Guangdong Provincial People’s Hospital, Guangdong Academy of Medical Sciences, Guangzhou, China; ^3^ WeiLun PET Center, Department of Nuclear Medicine, Guangdong Provincial People’s Hospital, Guangdong Academy of Medical Sciences, Guangzhou, China

**Keywords:** occult breast cancer, solitary splenic mass, metastasis, splenectomy, case report

## Abstract

Occult breast cancer, commonly presenting with axillary lymphadenopathy, is an extremely rare entity of breast cancer. Metastasis to the spleen as a single site is rarely seen and has been little reported in literature. Herein we described a case of a 60-year-old patient who presented with an asymptomatic solitary splenic mass 19 months after axillary lymph node dissection, regional radiotherapy, and systemic therapy. Laparoscopic splenectomy was performed, and histopathological examination confirmed metastasis from occult breast cancer. Then, the patient was administered with oral vinorelbine and dual-targeted treatment. With over 10 months of follow-up, there is no evidence of recurrence or metastasis of malignancy. To our knowledge, this study reports the first case of solitary splenic metastasis from occult breast cancer and highlights the importance of considering splenic metastasis as the only site of recurrence during follow-up of primary cancer, regardless of its rarity. If possible, splenectomy may be a therapeutic strategy.

## Introduction

Global cancer statistics suggest that breast cancer ranks first of all diagnosed cancers and represents the leading cause of cancer-related deaths among female patients encountered worldwide (1). Occult breast cancer, which generally presents as axillary lymph node involvement without identified primary breast lesions on clinical and radiologic examination, accounts for less than 1% of all breast cancer cases (2, 3). Although occult breast cancer with visceral metastasis to the bones, lungs, uterus, and liver is occasionally seen, metastasis to the spleen as a solitary lesion is a rare finding in those who have finished initial treatment. Herein we describe a case to illustrate the importance of considering occult breast cancer when interpreting solitary splenic metastasis.

## Case presentation

**Figure 1:**
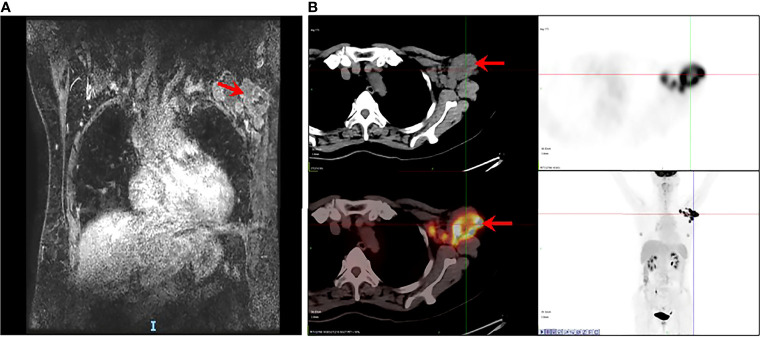
Occult breast cancer accompanied by metastasis in axillary lymph nodes. **(A)** MRI showing multiple enlarged and fused lymph nodes (arrow). **(B)** PET/CT showing multiple enlarged nodes on the deep muscles of the left axilla and the left anterior upper chest wall, radioactively concentrated (arrow) as well as with no evident metabolically active lesion in the bilateral breasts, remaining trunk, and brain.

A 60-year-old female was admitted to our hospital in June 2019 due to the presence of a left axillary mass. Dynamic contrast-enhanced magnetic resonance imaging (**Figure 1A**) was performed and confirmed multiple, ill-defined, irregular, heterogeneously enhanced nodes in the left axillary area, with the largest measuring 47 mm. The time–signal intensity curve obtained from axillary nodes showed early peak enhancement with rapid washout. Positron emission tomography with 2-deoxy-2-[fluorine-18] fluoro-D-glucose integrated with computed tomography (^18^F-FDG PET/CT) (**Figure 1B**) showed multiple enlarged lymph nodes in the deep muscles of the left axilla and the left anterior upper chest wall that were radioactively concentrated. Additionally, there were no evident metabolically active lesions in the bilateral breasts or on the ^18^F-FDG PET/CT of the remaining trunk and the brain.

Core-needle biopsy of lymph nodes was performed, and the pathological outcome revealed visible carcinoma in connective tissue. Immunohistochemistry (IHC) suggested ER-negative, PR-negative, CerbB2 3+, and Ki67 70%+. Next, the patient underwent 6 cycles of neoadjuvant therapy with TCbH (docetaxel/carboplatin/trastuzumab) followed by imaging evaluation, which showed 68% partial radiological response on targeted lymph node lesions according to the Response Evaluation Criteria in Solid Tumors (RECIST 1.1). The patient was identified as ycT0N1M0 in clinical stage and underwent left axillary lymph node dissection. The pathological findings from surgically resected specimens demonstrated that 7 of the 16 lymph nodes were positive and that vascular tumor thrombus existed. The pathological stage was deemed as ypT0N2M0. Postoperatively, targeted therapy was performed using trastuzumab, capecitabine-based adjuvant chemotherapy, and regional radiotherapy.

**Figure 2:**
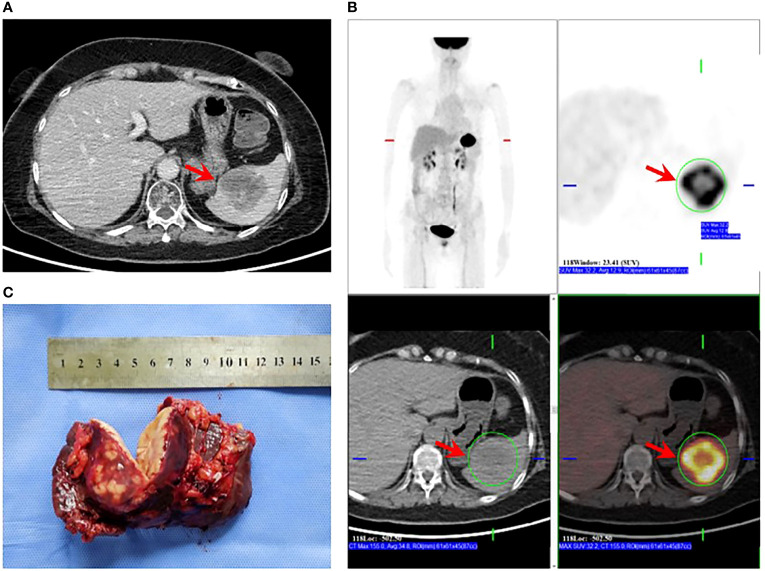
Splenectomy for solitary splenic metastasis. **(A)** CT showing a hypodense mass in the spleen that was heterogeneously enhanced after an enhanced scan (arrow). **(B)** PET/CT showing evidently increased FDG uptake of the splenic mass (arrow); SUVmax: 32.2; no sign of malignant tumor metabolism in the remaining body parts. **(C)** Specimen obtained post-splenectomy.

**Figure 3:**
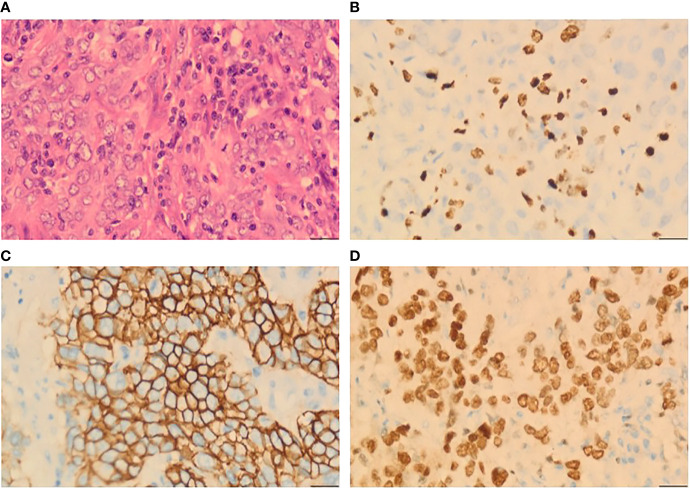
Histopathological analysis from a splenic lesion. **(A)** Microphotography showing poorly differentiated tumor cells (H&E, ×40). **(B)** 30% positive immunostaining for Ki67 (H&E, ×40). **(C)** Diffuse and strong positive for CerbB2 (H&E, ×40). **(D)** Diffuse strong nuclear staining for GATA3 (H&E, ×40).

After 19 months of regular follow-up, an abdominal computerized tomography (CT) scan (**Figure 2A**) revealed a single, hypodense, and heterogeneously enhanced lesion inside the spleen that was 54 × 51 mm in size. Further ^18^F-FDG PET/CT (**Figure 2B**) showed that FDG uptake was evidently increased, and the maximum of standardized uptake values (SUV) was 32.2. No manifestation of malignant tumor metabolism in the remaining body parts was found. At 2 weeks after pneumococcal vaccination, the patient underwent laparoscopic splenectomy on July 14, 2021 (**Figure 2C**). The postoperative pathological outcome was poorly differentiated carcinoma (**Figure 3**). Breast tumor metastasis was suspected, and IHC showed ER-negative, PR-negative, CerbB2 3+, Ki67 30%+, AR 10%+, GCDFP15-, GATA3 3+, and mammaglobin-.

The patient was in good physical condition after surgery and administered with oral vinorelbine and dual-targeted treatment (trastuzumab/pertuzumab). With over 10 months of follow-up visits in an oncology outpatient clinic, no evidence of recurrence or metastasis has been observed thus far.

## Discussion

Occult breast cancer, which manifests as clinically recognizable axillary lymph node metastases in the absence of primary breast lesions or distant disease on clinical and radiological examination, is a very rare entity of breast cancer (4). The most common sites of distant metastasis for invasive ductal carcinoma are the bone, liver, lung, lymph nodes, and brain (5, 6). Splenic metastases are extremely rare and are mostly diagnosed in the context of multivisceral metastatic cancer at the terminal stage of the disease or at autopsy. To the best of our knowledge, there have been three cases of breast cancer with solitary splenic metastasis (7–9) (**Table 1**). Only one case did not indicate a treatment strategy. The patient who presented with clinical manifestations and underwent splenectomy was in good health at 6 months of follow-up, but the survival outcome was not provided for another patient treated with chemotherapy. Notably, none of the metastatic lesions was from occult breast cancer. Upon review of the literature, this case was the first to present splenic metastasis as a solitary lesion after the initial treatment of occult breast cancer.

**Table 1:** Cases published in the literature of solitary splenic metastasis from breast cancer.

Reference	Publication year	Age	Breast cancer type	Symptoms	Imaging findings	Tumor size (cm)	Treatment	Prognosis
Iype *et al.* (8)	2002	54	IDC	Left hypochondrial pain and mild fever	CT: an enlarged spleen containing a well-circumscribed solitary hypodense area	5	Splenectomy	6 months, no recurrence or metastasis
Iga *et al.* (7)	2009	54	IDC	Asymptomatic	PET/CT: a discrete splenic metastasis	NA	Chemotherapy	NA
Sufficool *et al.* (9)	2012	48	IDC	Asymptomatic	CT: an isolated hypodense lesion within the anterior portion of the spleen MRI: an isolated lesion in the anterolateral aspect of the spleen	1.7	NA	NA

IDC, invasive ductal carcinoma; CT, computerized tomography; PET/CT: positron emission tomography/computed tomography; MRI, magnetic resonance imaging; NA, not available.

Different hypotheses have been proposed by various researchers to explain the rarity of splenic metastases (10). According to the mechanical factors, the constant blood flow, the evident acute angulation of the splenic artery branching from the celiac artery, and the rhythmic contractions of the splenic capsule may impede tumor implantation and growth in the spleen (11). Anatomically, the scarcity of lymphatic vessels in the spleen prevents the metastatic tumor cells from being transported into the spleen. In addition, the inhibitory effect of the splenic microenvironment, such as its high density of immune system cells, its role in “immune surveillance”, and its high concentration of angiogenesis inhibition factor, contributes to a low possibility of metastases (8, 12).

Accurately distinguishing non-neoplastic lesions, primary splenic lesions, and splenic metastases is crucial for clinicians to make therapeutic decisions. Notably, solitary splenic metastasis is mostly asymptomatic or sometimes occurs in association with nonspecific clinical manifestations, such as abdominal pain, fever, or weight loss. Notwithstanding image resolution and improved techniques such as computed tomography scanning, the clinical diagnosis of splenic metastases seems to be largely dependent on patient history. A solitary splenic mass without the context of active cancerous disease is more suggestive of a primary splenic lesion such as lymphoma, hemangioma, or infectious lesion. Conversely, in a patient with any prior or present history of malignancy, there is a need to rule out metastasis.

Indeed a so-called oligo-recurrence disease, characterized by a single/few detectable metastatic lesions existing after curative therapy for primary lesions, can be considered in this patient (13). Previous studies have revealed that selected patients with oligo-recurrent diseases may benefit from locally aggressive therapy compared with systemic management alone, especially when surgical resection offers the benefits of diagnostic confirmation of histology and therapeutic excision (14, 15). According to the 5th ESO-ESMO international consensus guidelines, 78% of experts supported that limited number and size of metastatic lesions may be potentially amenable for local therapy aimed at achieving a complete remission status (16). The study by Friedel *et al.* also demonstrated favorable survival in patients who underwent surgical resection of limited-volume pulmonary metastases (17). Therefore, even though no clear guidelines have been formulated thus far on the optimal management of the spleen-only metastasis in occult breast cancer because of its low incidence and unique clinical manifestations, we utilized the available retrospective data to synthesize therapeutic decisions and found that, for the treatment of solitary splenic metastasis, which is associated with a low incidence of complications and potential long-term survival benefits, splenectomy is a reasonable strategy (18). However, further studies are still needed to establish standardized recommendations for the management of this rare entity.

To date, the pattern of distant metastasis in occult breast cancer compared with that in invasive ductal carcinoma remains relatively unclear. Even if the primary tumor was successfully treated many years earlier, the possibility of splenic metastasis as the only site of recurrence should be taken into consideration during disease progression. Awareness of this unusual phenomenon would help in early diagnosis and treatment with consequent influence on patient management.

## Concluding remarks

Although extremely rare, the appearance of a splenic mass in a patient with a history of occult breast cancer should enable the consideration for a potential metastasis, and surgical resection may be a therapeutic strategy for patients with solitary splenic metastasis to prolong overall survival.

## Data availability statement

The original contributions presented in the study are included in the article/supplementary material. Further inquiries can be directed to the corresponding author.

## Ethics statement

Written informed consent was obtained from the patient for publication of this case report and accompanying images.

## Author contributions

YC and LZ were mainly responsible for article writing. TS performed the image acquisition. M-YC and JZ were responsible for the consent from the patient and ethics committee. KW was in charge of all study procedures. All authors contributed to the article and approved the submitted version.

## Conflict of interest

The authors declare that the research was conducted in the absence of any commercial or financial relationships that could be construed as a potential conflict of interest.

## Publisher’s note

All claims expressed in this article are solely those of the authors and do not necessarily represent those of their affiliated organizations, or those of the publisher, the editors and the reviewers. Any product that may be evaluated in this article, or claim that may be made by its manufacturer, is not guaranteed or endorsed by the publisher.

## References

[1] Bray F Ferlay J Soerjomataram I Siegel RL Torre LA Jemal A . Global cancer statistics 2018: Globocan estimates of incidence and mortality worldwide for 36 cancers in 185 countries. CA Cancer J Clin (2018) 68(6):394–424. doi: 10.3322/caac.21492 30207593

[2] Rueth NM Black DM Limmer AR Gabriel E Huo L Fornage BD . Breast conservation in the setting of contemporary multimodality treatment provides excellent outcomes for patients with occult primary breast cancer. Ann Surg Oncol (2015) 22(1):90–5. doi: 10.1245/s10434-014-3991-0 25249256

[3] Macedo FI Eid JJ Flynn J Jacobs MJ Mittal VK . Optimal surgical management for occult breast carcinoma: A meta-analysis. Ann Surg Oncol (2016) 23(6):1838–44. doi: 10.1245/s10434-016-5104-8 26832884

[4] Oualla K Elm’rabet F Arifi S Mellas N Melhouf MA Bouhafa T . Occult primary breast cancer presenting with axillary nodal metastasis: Report of 3 cases. J Clin Gynecol Obstet (2012) 1(4-5):85–8. doi: 10.4021/jcgo.v1i4-5.42

[5] Lam KY Tang V . Metastatic tumors to the spleen: A 25-year clinicopathologic study. Arch Pathol Lab Med (2000) 124(4):526–30. doi: 10.5858/2000-124-0526-MTTTS 10747308

[6] Wang R Zhu Y Liu X Liao X Niu L . The clinicopathological features and survival outcomes of patients with different metastatic sites in stage iv breast cancer. BMC Cancer (2019) 19(1):1091. doi: 10.1186/s12885-019-6311-z 31718602 PMC6852913

[7] Iga AM Robertson JH Al-Hejazi M . A diagnostic mishap: Solitary splenic carcinomatous metastases from the breast. Breast J (2009) 15(6):660–1. doi: 10.1111/j.1524-4741.2009.00808.x 19686227

[8] Iype S Akbar MA Krishna G . Isolated splenic metastasis from carcinoma of the breast. Postgrad Med J (2002) 78(917):173–4. doi: 10.1136/pmj.78.917.173 PMC1742304 11884703

[9] Sufficool K Wang J Doherty S . Isolated splenic metastasis from carcinoma of the breast: A case report. Diagn Cytopathol (2013) 41(10):914–6. doi: 10.1002/dc.22860 22644993

[10] Piura B Rabinovich A Apel-Sarid L Shaco-Levy R . Splenic metastasis from endometrial carcinoma: Report of a case and review of literature. Arch Gynecol Obstet (2009) 280(6):1001–6. doi: 10.1007/s00404-009-1039-7 19306010

[11] Comperat E Bardier-Dupas A Camparo P Capron F Charlotte F . Splenic metastases: Clinicopathologic presentation, differential diagnosis, and pathogenesis. Arch Pathol Lab Med (2007) 131(6):965–9. doi: 10.5858/2007-131-965-SMCPDD 17550328

[12] Baranyay F . Case report: Diffuse splenic metastasis of occult breast cancer with incompatible blood group antigenic determinants. Acta Histochem (2009) 111(4):343–8. doi: 10.1016/j.acthis.2008.11.015 19201455

[13] Niibe Y Hayakawa K . Oligometastases and oligo-recurrence: The new era of cancer therapy. Jpn J Clin Oncol (2010) 40(2):107–11. doi: 10.1093/jjco/hyp167 PMC2813545 20047860

[14] Schizas D Lazaridis II Moris D Mastoraki A Lazaridis LD Tsilimigras DI . The role of surgical treatment in isolated organ recurrence of esophageal cancer-a systematic review of the literature. World J Surg Oncol (2018) 16(1):55. doi: 10.1186/s12957-018-1357-y 29540179 PMC5853115

[15] Ohkura Y Shindoh J Ueno M Iizuka T Udagawa H . Clinicopathologic characteristics of oligometastases from esophageal cancer and long-term outcomes of resection. Ann Surg Oncol (2020) 27(3):651–9. doi: 10.1245/s10434-019-08175-0 31898096

[16] Cardoso F Paluch-Shimon S Senkus E Curigliano G Aapro MS André F . 5th eso-esmo international consensus guidelines for advanced breast cancer (Abc 5). Ann Oncol (2020) 31(12):1623–49. doi: 10.1016/j.annonc.2020.09.010 PMC7510449 32979513

[17] Friedel G Pastorino U Ginsberg RJ Goldstraw P Johnston M Pass H . Results of lung metastasectomy from breast cancer: Prognostic criteria on the basis of 467 cases of the international registry of lung metastases. Eur J Cardiothorac Surg (2002) 22(3):335–44. doi: 10.1016/s1010-7940(02)00331-7 12204720

[18] Sauer J Sobolewski K Dommisch K . Splenic metastases–not a frequent problem, but an underestimate location of metastases: Epidemiology and course. J Cancer Res Clin Oncol (2009) 135(5):667–71. doi: 10.1007/s00432-008-0502-3 PMC12160167 18936972

